# Work Environment Factors and Subjective Well-Being Among Healthcare Professionals: A Structural Equation Modeling Study with Mediation Analysis

**DOI:** 10.3390/ijerph23080976

**Published:** 2026-07-28

**Authors:** Evija Nagle, Maksims Zolovs, Anna Nyberg, Iluta Skrūzkalne, Ieva Saukuma, Andrejs Ivanovs, Ieva Reine

**Affiliations:** 1 Statistics Unit, Riga Stradiņš University, LV-1007 Riga, Latvia; 2 Institute of Life Sciences and Technology, Daugavpils University, LV-5401 Daugavpils, Latvia; 3 Department of Public Health and Caring Sciences, Uppsala University, SE-751 22 Uppsala, Sweden; 4 Faculty of Social Sciences, Riga Stradiņš University, LV-1007 Riga, Latvia

**Keywords:** psychosocial work environment, subjective well-being, healthcare professionals, structural equation modeling, mediation analysis, job demands–resources model

## Abstract

**Highlights:**

**Public health relevance—How does this work relate to a public health issue?**

- Healthcare professionals’ subjective well-being is closely related to workforce capacity, quality of care, patient safety, and the sustainability of healthcare systems.
- Psychosocial work environment factors, including job demands, autonomy, recognition, and occupational strain, represent modifiable organizational determinants of healthcare workforce well-being.

**Public health significance—Why is this work of significance to public health?**

- Healthcare systems increasingly face workforce shortages, high workloads, emotional strain, and burnout, making the protection of healthcare professionals’ well-being a public health priority.
- This study identifies psychosocial work environment factors associated with subjective well-being among healthcare professionals in Latvia, contributing evidence from a Central and Eastern European healthcare context.

**Public health implications—What are the key implications or messages for practitioners, policymakers, and/or researchers in public health?**

- Healthcare organizations should prioritize strategies that strengthen job satisfaction, recognition, autonomy, and work control while reducing occupational distress and strain.
- Workforce well-being interventions and public health policies should address both structural work environment conditions and motivational factors, while recognizing healthcare professionals’ well-being as an indicator of healthcare system sustainability and service quality.

**Abstract:**

Background: Healthcare professionals work in demanding psychosocial environments that may affect their subjective well-being and, consequently, the performance and sustainability of healthcare systems. Understanding how job demands and job resources are associated with healthcare professionals’ well-being is important for developing organizational strategies that support workforce health and service quality. Methods: A cross-sectional quantitative study was conducted among 566 healthcare professionals in Latvia. Psychosocial work environment factors were assessed using a multidimensional instrument based on the Job Demands–Resources (JD-R) model, while subjective well-being was measured using the WHO-5 Well-Being Index. A comprehensive multiple-predictor mediation SEM was estimated using the WLSMV estimator. The primary covariate-adjusted model was based on 561 complete cases and simultaneously included all four psychosocial predictors, Work Engagement and Vitality as the mediator, subjective well-being as the outcome, and age, work experience, and professional position as covariates. Results: The comprehensive model demonstrated good fit. Job Satisfaction and Reward showed significant positive direct and indirect associations with subjective well-being and the strongest total association. Occupational Distress and Strain showed significant negative indirect and total associations, whereas their direct associations were not statistically significant. Autonomy and Work Control showed a significant positive indirect association and a significant positive total association, while its direct association was not statistically significant. Organizational Trust and Support demonstrated a positive indirect association but a negative direct association, whereas its total association was not statistically significant, indicating inconsistent mediation. Conclusions: The findings suggest that psychosocial work environment factors are meaningfully associated with subjective well-being among healthcare professionals. Job Satisfaction and Reward, Autonomy and Work Control, and Occupational Distress and Strain appear to be particularly relevant factors. Work Engagement and Vitality may serve as an indirect pathway linking selected work environment factors to subjective well-being; however, given the cross-sectional design, these findings should be interpreted as statistical associations rather than evidence of causal mediation.

## 1. Introduction

The healthcare system is one of the most socially significant sectors, characterized by high psychological, emotional, and organizational demands. Healthcare professionals operate in dynamic and often stressful work environments that require rapid decision-making, high levels of responsibility, and continuous interaction with patients in complex clinical situations. Under such conditions, the subjective well-being of healthcare professionals becomes critically important, as it affects not only employees’ health and work capacity but also the quality of healthcare services and patient safety [1,2,3].

The work environment of healthcare professionals is inherently demanding, involving high workload, emotional strain, and frequent exposure to challenging situations, all of which can significantly affect psychological functioning and professional performance. Empirical studies indicate that lower levels of well-being among healthcare professionals are associated with increased risk of burnout, reduced job satisfaction, and decreased quality of patient care [1,4,5,6]. Therefore, subjective well-being in the healthcare sector is considered a key indicator of both employee health and the overall effectiveness of healthcare systems.

Subjective well-being is an individual’s subjective evaluation of psychological functioning, emotional state, and life satisfaction [7,8]. In this study, the term “subjective well-being” refers to subjective well-being in the work context, emphasizing emotional, cognitive, and functional aspects of the work environment. The World Health Organization emphasizes that well-being encompasses not only the absence of illness but also positive psychological functioning and the ability to cope effectively with everyday life [9]. Similarly, the Organization for Economic Co-operation and Development conceptualizes well-being as a multidimensional construct that includes subjective, economic, and work-related aspects, thereby highlighting the importance of broader contextual factors [10].

In addition to these conceptual frameworks, increasing attention has been given to European Union sustainability regulations, particularly the Corporate Sustainability Reporting Directive (CSRD), which emphasizes the social dimension of organizational performance. Within the CSRD framework, employee well-being, safe working conditions, and psychosocial work environments are recognized as essential sustainability indicators that directly influence long-term organizational effectiveness and societal well-being [11]. In the healthcare sector, this perspective is particularly relevant, as the subjective well-being of healthcare professionals is closely linked to service quality, patient safety, and system resilience. Thus, the psychosocial work environment and employee well-being should be viewed not only as individual experiences but also as strategically important organizational and public health issues.

The work environment is a key determinant of healthcare professionals’ subjective well-being. Job demands, such as high work intensity, emotional strain, and time pressure, can negatively affect employees’ psychological functioning. In contrast, job resources, including social support, opportunities for professional development, and autonomy, can enhance motivation, engagement, and well-being [4,12,13,14,15]. This perspective is consistent with the Job Demands–Resources (JD-R) model, which explains how work environment factors influence employee well-being through two primary processes: the health impairment process and the motivational process [13,16].

In the present study, work environment factors are operationalized based on an empirically validated multidimensional structure comprising five key latent constructs. Occupational Distress and Strain are conceptualized as indicators of job demands, reflecting physical and emotional workload. In contrast, Organizational Trust and Support, Autonomy and Work Control, and Job Satisfaction and Reward represent job resources that foster motivation and positive work experiences [12,14]. In addition, Work Engagement and Vitality are defined as central psychological mechanisms that mediate the relationship between work environment factors and subjective well-being, reflecting employees’ energy, involvement, and professional activity [6,17]. This approach integrates both the health impairment process associated with job demands and the motivational process driven by job resources within a unified analytical framework, thereby ensuring a theoretically grounded application of the JD-R model in the healthcare context [13,16].

Despite the growing body of research on healthcare professionals’ well-being, several important research gaps remain. First, most studies focus on direct relationships between work environment factors and psychological outcomes. In contrast, mediating mechanisms that explain these relationships are examined less frequently [6,14]; research in the healthcare sector has predominantly focused on negative outcomes, such as stress and burnout. At the same time, positive dimensions of subjective well-being have received comparatively less attention [1,18,19]. Third, there are still a limited number of studies employing empirically validated, context-specific instruments and advanced analytical approaches, such as structural equation modeling, which allow for the simultaneous examination of multiple interrelated relationships.

Based on the identified research gaps, this study aims to examine the direct and indirect associations between psychosocial work environment factors and healthcare professionals’ subjective well-being, with a particular focus on the mediating roles of work engagement and vitality, using a structural equation modeling approach. The study analyses both direct and indirect relationships between work environment factors and subjective well-being, thereby providing a comprehensive understanding of the associations underlying well-being in healthcare settings.

### 1.1. Work Environment Factors and Subjective Well-Being

In this study, subjective well-being is operationalized using the WHO-5 Well-Being Index, which is a widely used and validated instrument for assessing positive psychological well-being [19]. Based on the Job Demands–Resources (JD-R) theoretical framework, work environment factors are conceptualized as key psychosocial determinants that influence employee well-being through both the health impairment and motivational processes [13,16].

Occupational Distress and Strain are conceptualized as indicators of job demands, reflecting increased workload, emotional strain, and physical fatigue. According to the JD-R model, high job demands activate the health impairment process, which negatively affects employees’ psychological functioning and reduces subjective well-being [4,6,16]. Empirical studies consistently demonstrate that higher levels of distress in healthcare settings are associated with lower subjective well-being [1].

Organizational Trust and Support reflect employees’ perceptions of organizational support, trust in management, and the overall social climate in the workplace. It is considered a key job resource that enhances psychological safety, a sense of belonging, and emotional stability, thereby positively influencing subjective well-being [14,18].

Autonomy and Work Control represent employees’ ability to influence their work processes and make independent decisions. Autonomy is a central job resource that promotes intrinsic motivation, professional effectiveness, and psychological well-being [13,14].

Job Satisfaction and Reward reflect employees’ satisfaction with their work, as well as their perceptions of compensation, recognition, and professional appreciation. This factor functions as an important motivational resource and is associated with more positive emotional states and higher levels of subjective well-being [15,18].

Overall, based on the JD-R theoretical framework, job demands and job resources influence subjective well-being in opposite directions: job demands act as risk factors, whereas job resources promote positive psychological outcomes and function as protective factors.

Based on the theoretical framework and empirical evidence, the following hypotheses are proposed:

**H1.**  *Occupational Distress and Strain are negatively associated with healthcare professionals’ subjective well-being.*

**H2a.**  *Organizational Trust and support are positively associated with healthcare professionals’ subjective well-being.*

**H2b.**  *Autonomy and Work Control are positively associated with healthcare professionals’ subjective well-being.*

**H2c.**  *Job Satisfaction and rewards are positively associated with healthcare professionals’ subjective well-being.*

To further explain these relationships, the role of work engagement and vitality as mediators is examined.

### 1.2. Mediating Mechanisms

Within the Job Demands–Resources (JD-R) theoretical framework, the effects of work environment factors on employees’ subjective well-being are not limited to direct relationships; underlying psychological mechanisms also explain them. The JD-R model proposes that job demands and job resources influence well-being through two interrelated processes: the health impairment process and the motivational process [13,16].

A central mechanism representing the motivational process is work engagement, defined as a positive, work-related psychological state characterized by energy, involvement, and professional vitality [6,17]. In this study, Work Engagement and Vitality were conceptualized as potential mediators linking psychosocial work environment factors with healthcare professionals’ subjective well-being. These mediators were selected on the basis of the JD-R theoretical framework and previous empirical evidence rather than on the basis of preliminary correlations observed in the present dataset.

According to the JD-R model, job resources such as Organizational Trust and Support, Autonomy and Work Control, and Job Satisfaction and Reward play a crucial role in activating the motivational process. These resources provide the psychological and organizational conditions necessary to foster higher levels of work engagement. Increased levels of job resources are associated with greater employee energy, involvement, and professional activity, which in turn contribute to higher levels of subjective well-being [13,14].

In contrast, job demands, represented in this study by Occupational Distress and Strain, may negatively affect work engagement by contributing to exhaustion and reducing employees’ energy and motivation. This reflects the activation of the health impairment process, which, in turn, indirectly diminishes subjective well-being [6,16].

Thus, work engagement functions as a central mediating mechanism through which both job demands and job resources influence healthcare professionals’ subjective well-being. Methodologically, a statistically significant zero-order correlation between a proposed mediator and the outcome is not a prerequisite for testing an indirect effect. Mediation is evaluated through the product of the path from the predictor to the mediator and the path from the mediator to the outcome. Therefore, a statistically significant indirect association may occur even when the overall bivariate association is weak or non-significant, particularly when direct and indirect pathways operate in opposite directions or when suppression effects are present.

Empirical evidence consistently supports the mediating role of work engagement in the relationships between job resources and positive work-related outcomes, and between job demands and psychological well-being [6,14].

Accordingly, the mediation hypotheses were retained because they were theoretically specified before the statistical analyses and were evaluated directly within the SEM framework using estimated indirect effects and their confidence intervals. However, because the present study used a cross-sectional design, the resulting mediation estimates are interpreted as statistical indirect associations rather than evidence of temporal or causal mechanisms.

Based on the JD-R theoretical framework and empirical evidence, the following hypotheses are proposed:

**H3a.**  *Work Engagement and Vitality mediate the relationship between Occupational Distress and Strain and healthcare professionals’ subjective well-being.*

**H3b.**  *Work Engagement and Vitality mediate the relationship between Organizational Trust and Support and healthcare professionals’ subjective well-being.*

**H3c.**  *Work Engagement and Vitality mediate the relationship between Autonomy and Work Control and healthcare professionals’ subjective well-being.*

**H3d.**  *Work Engagement and Vitality mediate the relationship between Job Satisfaction and Reward and healthcare professionals’ subjective well-being.*

Based on these relationships, a conceptual model is developed and tested using SEM.

### 1.3. Conceptual Model and Rationale for SEM

Based on the Job Demands–Resources (JD-R) theoretical framework and the empirical evidence outlined above, this study proposes a conceptual model that integrates psychosocial work environment factors and subjective well-being within a unified analytical framework [12,13,16]. The model assumes that job demands and job resources influence healthcare professionals’ subjective well-being both directly and indirectly through work engagement as a central mediating mechanism [14,17].

Specifically, Occupational Distress and Strain are conceptualized as indicators of job demands that negatively affect subjective well-being and simultaneously reduce work engagement by activating the health impairment process [4,16]. In contrast, Organizational Trust and Support, Autonomy and Work Control, and Job Satisfaction and Reward are defined as job resources that enhance work engagement and positively influence subjective well-being by activating the motivational process [13,14,18]. Work Engagement and Vitality function as the central mediators in the model, explaining the indirect effects of work environment factors on subjective well-being [6,17].

Thus, the proposed model reflects both the health impairment process (job demands → negative outcomes) and the motivational process (job resources → positive outcomes) as defined within the JD-R framework, while integrating mediation mechanisms within a single structural model [13,14].

Given the conceptual complexity of the model, which includes multiple latent constructs and simultaneous direct and indirect relationships, structural equation modeling (SEM) is employed to test the proposed hypotheses. This approach is particularly suitable for testing complex theoretical models involving multiple simultaneous relationships and mediation effects. SEM enables the simultaneous estimation of relationships among latent variables, accounts for measurement error, and allows testing of mediation effects within a single analytical framework, making it particularly suitable for analyzing complex JD-R-based models [20,21].

Furthermore, SEM allows simultaneous examination of multiple interrelated pathways, providing a comprehensive assessment of the effects of job demands and job resources on subjective well-being, as well as the roles of mediating mechanisms. This approach offers deeper insight into the psychosocial processes underlying healthcare professionals’ well-being in the work environment [21].

## 2. Materials and Methods

### 2.1. Study Design

A cross-sectional quantitative study design was employed to empirically examine the relationships between psychosocial work environment factors and healthcare professionals’ subjective well-being. The cross-sectional approach was selected because it allows analysis of interrelationships among multiple psychosocial variables at a single point in time, enabling identification of statistically significant associations between work environment characteristics and well-being outcomes [22,23].

The study was conducted as an observational study without any intervention in the work environment or participants’ behavior. This approach ensures high external validity and enhances the applicability of the findings to real-world healthcare settings.

### 2.2. Participants and Sampling

The study sample consisted of healthcare professionals employed in Latvia’s healthcare sector, including both public and private institutions. The target population comprised healthcare professionals employed in Latvian hospitals and directly involved in patient care. The accessible population consisted of eligible healthcare professionals employed in the 13 hospitals participating in the study. Participants were recruited using a non-probability convenience sampling approach, which is commonly applied in healthcare research where access to the target population is limited [24].

The inclusion criteria were: (1) employment as a healthcare professional, (2) direct involvement in patient care, and (3) voluntary consent to participate in the study. Respondents with a substantial proportion of missing data (>20%) were excluded from the analysis to ensure data quality and the accuracy of the results [25].

Sample size adequacy was evaluated using the observation-to-parameter ratio recommended for structural equation modeling. The most complex comprehensive model included 51 freely estimated parameters. Based on the commonly recommended ratio of 10 observations per estimated parameter, the minimum required sample size was 510 participants. The primary complete-case analytical sample comprised 561 participants, corresponding to an achieved observation-to-parameter ratio of 11.00:1. Thus, the available sample size was considered adequate for estimating the proposed structural model [20,26].

### 2.3. Measures

#### 2.3.1. Dependent Variable

Healthcare professionals’ subjective well-being was assessed using the WHO-5 Well-Being Index, a widely used and validated instrument for measuring positive psychological well-being [19]. The WHO-5 consists of 5 items that reflect emotional well-being over the past 2 weeks. The WHO-5 score was calculated as the sum of the item scores, with higher total scores indicating greater subjective well-being.

#### 2.3.2. Independent Variables

Psychosocial work environment factors were assessed using the author-developed Healthcare Professionals Psychosocial Well-Being Scale (HP-PSWB). The English and Latvian versions of the HP-PSWB scale are provided in Supplementary Materials Files S1 and S2, respectively. The instrument was developed based on an integrated theoretical framework that combines the Job Demands–Resources (JD-R) model [13,16], the World Health Organization (WHO) well-being framework [9], and the Organization for Economic Co-operation and Development (OECD) multidimensional well-being approach [27].

The instrument was designed to assess key psychosocial characteristics of the healthcare work environment, including job demands, job resources, organizational support, autonomy, reward, and work-related engagement. The scale was developed and psychometrically validated using a split-sample approach. First, exploratory factor analysis (EFA) was conducted to identify the underlying factor structure, followed by confirmatory factor analysis (CFA) to validate the measurement model. The total sample of 566 participants was randomly divided into two non-overlapping subsamples. Exploratory factor analysis was conducted in the first subsample (*n* = 260), whereas confirmatory factor analysis was conducted in the second independent subsample (*n* = 306).

The primary item-level EFA loadings and standardized CFA factor loadings for the retained 35-item HP-PSWB scale are presented in Table 1 and Table 2, respectively.

The final optimized five-factor model demonstrated an acceptable-to-good fit to the data: χ^2^ = 1382.49, *p* < 0.001; CFI = 0.925; TLI = 0.918; RMSEA = 0.071; and SRMR = 0.080. These results supported the factorial validity of the measurement model. As shown in Table 2, standardized factor loadings ranged from 0.409 to 0.847, with the majority exceeding the recommended threshold of 0.50, indicating adequate indicator reliability.

Internal consistency of the constructs ranged from acceptable to high. Cronbach’s alpha values ranged from 0.66 to 0.91, while composite reliability values ranged from 0.64 to 0.92.

Convergent validity was evaluated using average variance extracted (AVE), with values ranging from 0.33 to 0.59. Although some AVE values were below the recommended threshold of 0.50, the corresponding CR values exceeded 0.60. However, CR values above 0.60 were interpreted as providing partial support for construct reliability rather than as fully compensating for AVE values below 0.50 [21]. Accordingly, the convergent validity of Organizational Trust and Support and Autonomy and Work Control should be considered limited and in need of further confirmation.

Discriminant validity was assessed using the heterotrait–monotrait ratio of correlations (HTMT). All HTMT values ranged from 0.183 to 0.690 and remained below the conservative threshold of 0.85, supporting satisfactory discriminant validity.

Autonomy and Work Control demonstrated Cronbach’s α = 0.662, CR = 0.644, and AVE = 0.328, with standardized factor loadings ranging from 0.481 to 0.839. The construct was retained because it represents a theoretically central job resource within the JD-R framework and because its five items capture complementary aspects of autonomy, including workday planning, influence over workload, participation in decision-making, choice of work methods, and autonomy in task performance. The CR value marginally exceeded the recommended minimum threshold of 0.60, and the HTMT results supported the construct’s discriminant distinctiveness. Nevertheless, the relatively low AVE and Cronbach’s alpha indicate limited item homogeneity and convergent validity. Therefore, structural associations involving Autonomy and Work Control should be interpreted cautiously. Further independent validation and possible refinement of the lower-loading items. Further independent validation and possible refinement of the lower-loading items, particularly A10 (loading = 0.481) and A7 (loading = 0.494), are recommended.

The final measurement model comprised five latent constructs: Job Satisfaction and Reward (9 items; α = 0.91), Work Engagement and Vitality (8 items; α = 0.83), Occupational Distress and Strain (6 items; α = 0.79), Organizational Trust and Support (7 items; α = 0.78), and Autonomy and Work Control (5 items; α = 0.66).

Items were rated on a four-point Likert-type agreement scale: 1 = strongly disagree, 2 = somewhat disagree, 3 = somewhat agree, and 4 = agree. An additional response option, “prefer not to answer/not applicable,” was provided to allow respondents to indicate that a statement was not relevant to their work situation or that they did not wish to answer. This response option was not treated as part of the ordinal agreement scale and was coded as missing for scale scoring and statistical analysis.

For each construct, scale scores were calculated as the mean of the corresponding items, provided that a sufficient number of valid responses were available. Higher scores indicated higher levels of the respective construct. To ensure consistency in the direction of interpretation, reverse coding was applied where necessary. In total, 11 items were reverse-coded across three constructs: Job Satisfaction and Reward (3 items), Work Engagement and Vitality (2 items), and Occupational Distress and Strain (6 items). No reverse-coded items were included in the Organizational Trust and Support or Autonomy and Work Control constructs.

After the factor structure had been identified in the EFA subsample (*n* = 260) and evaluated in the separate CFA subsample (*n* = 306), the full sample (*N* = 566) was carried forward to the structural analyses. The comprehensive covariate-adjusted SEM was estimated using complete cases (*N* = 561), because five participants had missing data for one or more covariates.

In the structural equation modeling (SEM) framework, psychosocial work environment factors were modeled as latent variables, with each construct represented by its corresponding observed indicators.

### 2.4. Covariates

Age, work experience, and professional position were included as covariates in the comprehensive structural model. These variables were selected based on their well-established theoretical and empirical associations with professional experience and subjective well-being, as well as their potential influence on the perception of the psychosocial work environment [4,13,14]. Professional position was included as a categorical covariate because occupational groups may differ in job demands, autonomy, workload, and psychosocial exposures. Physicians were used as the reference category.

Other sociodemographic and work-related variables were not included in the structural model to maintain model parsimony and avoid unnecessary increases in the number of estimated parameters, which could reduce model stability and interpretability [28].

In particular, gender was not included as a covariate due to the highly unbalanced sample distribution (89.9% female, 8.5% male), which limits statistical power and may yield unstable parameter estimates [29]. Under such conditions, including gender in the model could compromise the model’s reliability and interpretability. To evaluate the robustness of the findings in relation to the predominance of female participants, the comprehensive model was additionally re-estimated in a female-only sensitivity analysis.

Other variables, including healthcare institution type, workload, and education level, were not included because they were not central to the prespecified theoretical model and their inclusion would have substantially increased model complexity.

Thus, the selected covariates enabled control of the most relevant aspects of individual professional experience while maintaining a parsimonious, stable, and interpretable model. Age, work experience, and professional position were included in the SEM analysis as observed covariates.

### 2.5. Data Collection Procedure

Data were collected between January and March 2026 using an anonymous, online, self-administered questionnaire. The survey was distributed to healthcare professionals employed in 13 hospitals across Latvia.

In each participating hospital, a human resources representative disseminated the survey invitation to eligible healthcare professionals through institutional work email addresses, internal WhatsApp groups, and other internal communication channels. Because each hospital managed the distribution process independently and did not record how many employees received or viewed the invitation, the total number of invited healthcare professionals could not be determined. Consequently, a conventional response rate could not be calculated, and potential non-response bias could not be directly assessed.

Before accessing the questionnaire, participants were provided with information about the purpose of the study, the voluntary nature of participation, the expected time required to complete the questionnaire, data processing procedures, anonymity, and confidentiality. Electronic informed consent was obtained before participants were granted access to the survey. No financial or other incentives were provided.

A total of 593 questionnaires were received. A prespecified data-quality criterion was applied, whereby questionnaires with more than 20% unanswered items were excluded. This criterion was used because substantial item-level missingness could prevent reliable calculation of scale scores and compromise the stability and interpretability of the structural equation modeling estimates. Twenty-seven questionnaires met this exclusion criterion and were removed, resulting in a full study sample of 566 participants.

### 2.6. Data Analysis

Statistical analyses were conducted using jamovi version 2.7.38 (The jamovi project, Sydney, Australia) and the SEMLj module version 1.2.5 (Marcello Gallucci and Sebastian Jentschke; https://semlj.github.io/, accessed on 26 July 2026) for structural equation modeling. Jamovi was selected because it supports latent-variable SEM, mediation analysis, and the WLSMV estimator, which is appropriate for ordinal and non-normally distributed data. The software also provides model-fit indices and estimates of direct and indirect associations required for the present analyses. Before the main analyses, data screening procedures were performed. These included assessing missing values, response distributions, and potential outliers. Missing data were examined at both the case and item levels. A total of 593 questionnaires were received. Of these, 27 questionnaires containing more than 20% unanswered items were excluded according to a prespecified data-quality criterion. This level of missingness did not allow reliable calculation of several scale scores and could compromise the stability and interpretability of the SEM estimates.

The remaining 566 questionnaires met the prespecified inclusion criterion and constituted the full study sample. However, five participants had missing data for one or more covariates included in the comprehensive adjusted SEM. Consequently, the primary structural model was estimated using complete cases (*N* = 561). No data imputation was performed.

Responses marked as “prefer not to answer/not applicable” were treated as missing values and were not included in scale score calculation or statistical modeling. No formal statistical test of the missing-data mechanism was conducted. Therefore, the mechanisms underlying missingness in the 27 excluded questionnaires and the missing covariate data in the five participants excluded from the adjusted SEM could not be determined [30].

Before computing scale scores, negatively worded items were reverse-coded where necessary to ensure that higher scores consistently reflected higher levels of the respective construct. For each psychosocial work environment factor, mean scores were calculated using only valid item responses [31]. Descriptive statistics, including median and interquartile range (IQR), were calculated for all study variables.

Normality was assessed using skewness, kurtosis, and the Shapiro–Wilk test. The results indicated that all study variables significantly deviated from a normal distribution (*p* < 0.001). Therefore, non-parametric correlation analysis was applied in the preliminary analysis, and Spearman’s rho coefficients were used to examine bivariate associations between study variables.

To test the study hypotheses, a comprehensive multiple-predictor mediation SEM was estimated. Occupational Distress and Strain, Organizational Trust and Support, Autonomy and Work Control, and Job Satisfaction and Reward were entered simultaneously as predictors of Work Engagement and Vitality, as well as subjective well-being. Direct paths from all four psychosocial predictors to subjective well-being and specific indirect paths through Work Engagement and Vitality were estimated within a single analytical framework. The model was adjusted for age, work experience, and professional position, with physicians serving as the reference category [20].

Given that the study variables were measured using ordinal Likert-type response categories and did not meet the assumption of normality, the model was estimated using the Weighted Least Squares Mean and Variance adjusted (WLSMV) estimator, which is appropriate for ordinal and non-normally distributed data [32].

Model fit was evaluated using multiple fit indices: the Comparative Fit Index (CFI) and Tucker–Lewis Index (TLI), with values ≥ 0.90 indicating acceptable fit, and the Root Mean Square Error of Approximation (RMSEA) and Standardized Root Mean Square Residual (SRMR), with values ≤ 0.08 indicating acceptable to good model fit [26,33].

Statistical indirect associations were assessed within the SEM framework and were considered statistically significant when their 95% confidence intervals did not include zero [34]. Direct, indirect, and total associations were estimated simultaneously. Given the cross-sectional study design, the indirect associations were interpreted as statistical pathways rather than evidence of temporal or causal mediation.

Given the marked predominance of female participants in the sample, a sensitivity analysis was conducted by re-estimating the comprehensive multiple-predictor mediation SEM among female healthcare professionals only. The same predictors, mediator, outcome, covariates, WLSMV estimator, and model specification as in the primary analysis were retained. Of the 509 female participants, four had missing data for one or more covariates and were excluded through complete-case analysis, resulting in a female-only analytical sample of 505 participants.

### 2.7. Ethical Considerations

The study was conducted in accordance with internationally recognized ethical principles for research involving human participants, including the Declaration of Helsinki [35], and complied with the data protection requirements of the European Union’s General Data Protection Regulation (GDPR) [36].

Before data collection, ethical approval was obtained from the Research Ethics Committee of Rīga Stradiņš University. The committee evaluated the study protocol, objectives, data collection procedures, and data protection measures. The study was approved at the meeting held on 11 September 2025, and permission to conduct the study was granted on 4 November 2025 (decision No. 2-PĒK-4/1084/2025).

Participation in the study was voluntary, and informed consent was obtained from all participants before completing the questionnaire. The survey was anonymous, ensuring participants’ anonymity and confidentiality. Data were processed in accordance with research ethics and data protection principles.

## 3. Results

### 3.1. Participant Characteristics

Most respondents were female (89.9%), while males accounted for 8.5% of the sample. In terms of employment setting, the largest proportion of participants were employed in public healthcare institutions (49.5%), followed by municipal institutions (27.7%) and private healthcare institutions (12.7%). The “Other” category (10.1%) included respondents with mixed employment arrangements (e.g., working simultaneously in multiple healthcare institutions or across different sectors), as well as those who did not specify their workplace.

Regarding professional roles, physicians constituted the largest group (42.8%), followed by nurses (30.6%) and physician assistants (8.3%). The “Other” category (11.8%) included physiotherapists, other rehabilitation professionals involved in functional assessment and treatment, laboratory personnel, and other healthcare professionals not classified within the primary categories.

Regarding workload, most respondents reported working 1.0 full-time equivalent (65.9%). A substantial proportion (18.9%) indicated working more than one full-time equivalent, suggesting an increased workload.

Smaller proportions of respondents reported working part-time, including 0.5 full-time equivalent (4.9%), 0.75 full-time equivalent (4.9%), and 0.25 full-time equivalent or less (0.9%). The “Other” category (4.5%) included respondents with non-standard employment arrangements, such as contractual work (e.g., service agreements with organizations), as well as those who did not specify their workload.

In terms of educational background, the largest proportion of respondents held a medical degree (41.0%), followed by a short-cycle higher education degree (31.6%). Smaller proportions had completed postgraduate medical residency training (7.1%) or held a doctoral degree (1.4%). The “Other” category (18.9%) included respondents with other or unspecified educational qualifications.

The full study sample comprised 566 participants. The primary covariate-adjusted SEM was estimated using 561 complete cases, as described in Section 2.6.

Descriptive characteristics of the sample are presented in Table 3.

### 3.2. Descriptive Statistics

Descriptive statistics for the study variables are presented in Table 4. Because the variables were measured on Likert-type scales, medians and interquartile ranges (IQRs) were reported.

The results indicated that median values for psychosocial work environment factors ranged from 2.83 to 3.13, while subjective well-being had a median of 16.00, suggesting moderate levels across the sample. The interquartile ranges indicated moderate variability, particularly for subjective well-being (IQR = 7.00), compared to narrower distributions observed for the work environment factors.

Skewness and kurtosis values suggested slight deviations from normality across all variables. This was further confirmed by the Shapiro–Wilk test, which indicated that all variables were significantly non-normal (*p* < 0.001).

### 3.3. Preliminary Analysis

#### Correlation Analysis

Given that the study variables were measured using Likert-type response categories and did not meet the assumption of normality, Spearman’s rank-order correlation analysis was conducted to examine bivariate associations between psychosocial work environment factors and healthcare professionals’ subjective well-being.

For interpreting correlation coefficients, Cohen’s conventional guidelines were applied: values around 0.10 indicate a small effect, values around 0.30 indicate a moderate effect, and values of 0.50 or higher indicate a large effect.

The results showed that healthcare professionals’ subjective well-being was significantly associated with several psychosocial factors in the work environment (Table 5). A large positive correlation was observed between subjective well-being and Job Satisfaction and Reward (r = 0.521, *p* < 0.001), as well as between subjective well-being and Autonomy and Work Control (r = 0.512, *p* < 0.001). Organizational Trust and Support also demonstrated a moderate positive association with subjective well-being (r = 0.451, *p* < 0.001).

In contrast, Occupational Distress and Strain showed a moderate negative association with subjective well-being (r = −0.442, *p* < 0.001), indicating that higher levels of work-related distress and strain were associated with lower subjective well-being.

Work Engagement and Vitality did not show a statistically significant bivariate association with subjective well-being (r = 0.010, *p* = 0.816). This finding suggests that, at the bivariate level, Work Engagement and Vitality was not directly associated with subjective well-being in the present sample. Therefore, its role was examined further in the structural and mediation analyses to determine whether it functioned as an indirect mechanism within the broader theoretical model.

Overall, the preliminary correlation analysis indicated that job resources were positively associated with subjective well-being, whereas job demands were negatively associated with subjective well-being. However, the absence of a significant bivariate correlation between the Work Engagement and Vitality construct and subjective well-being suggests that its role should be interpreted cautiously and within the context of the comprehensive structural model.

### 3.4. Comprehensive Multiple-Predictor Mediation Model

To examine the proposed direct and indirect associations while accounting for overlap among the psychosocial work environment factors, a comprehensive multiple-predictor mediation SEM was estimated. Occupational Distress and Strain, Organizational Trust and Support, Autonomy and Work Control, and Job Satisfaction and Reward were entered simultaneously as predictors of the mediator, Work Engagement and Vitality, and of subjective well-being. Direct paths from all four psychosocial predictors to subjective well-being were also estimated. The model was adjusted for age, work experience, and professional position, with physicians serving as the reference category. Of the 566 participants, five had missing data for one or more covariates and were excluded through complete-case analysis, resulting in an analytical sample of 561 participants.

The comprehensive model demonstrated good fit to the data: χ^2^(951) = 2504.95, *p* < 0.001; CFI = 0.944; TLI = 0.954; RMSEA = 0.054, 95% CI [0.051, 0.057]; and SRMR = 0.064. The model explained 60.7% of the variance in Work Engagement and Vitality and 57.5% of the variance in subjective well-being.

Regarding the direct paths to subjective well-being, Job Satisfaction and Reward was the only predictor showing a statistically significant association in the hypothesized positive direction (β = 0.440, *p* < 0.001), supporting H2c. Occupational Distress and Strain (β = −0.096, *p* = 0.101) and Autonomy and Work Control (β = 0.065, *p* = 0.216) were not significantly directly associated with subjective well-being after simultaneous adjustment for the other psychosocial predictors and covariates. Therefore, H1 and H2b were not supported at the direct-path level. Organizational Trust and Support showed a statistically significant negative direct association with subjective well-being (β = −0.160, *p* = 0.001), which was opposite to the hypothesized direction; therefore, H2a was not supported. However, the total associations of Occupational Distress and Strain and Autonomy and Work Control remained statistically significant in the expected directions, indicating that their associations with subjective well-being were primarily accounted for by the statistical indirect pathways through Work Engagement and Vitality.

Work Engagement and Vitality were positively associated with subjective well-being (β = 0.476, *p* < 0.001). Occupational Distress and Strain showed a significant negative association with Work Engagement and Vitality (β = −0.333, *p* < 0.001) and a significant negative indirect association with subjective well-being (B = −0.166, 95% CI [−0.235, −0.097], *p* < 0.001). Its direct association with subjective well-being was not statistically significant, whereas the total association remained negative and significant. This result supported H3a as a statistical indirect-only association.

Organizational Trust and Support were positively associated with Work Engagement and Vitality (β = 0.216, *p* < 0.001). However, the positive indirect association with subjective well-being (B = 0.281, 95% CI [0.138, 0.424], *p* < 0.001) was accompanied by a significant negative direct association (β = −0.160, *p* = 0.001), while the total association was not statistically significant. This opposing pattern indicated inconsistent mediation and did not provide clear support for H3b.

Autonomy and Work Control showed a significant positive association with Work Engagement and Vitality (β = 0.273, *p* < 0.001) and a significant positive indirect association with subjective well-being (B = 0.162, 95% CI [0.085, 0.238], *p* < 0.001). Its direct association was not statistically significant, while the total association remained positive and significant. Thus, H3c was supported as a statistical indirect-only association.

Job Satisfaction and Reward demonstrated the strongest positive association with Work Engagement and Vitality (β = 0.615, *p* < 0.001). Both its direct association with subjective well-being (β = 0.440, *p* < 0.001) and its indirect association through Work Engagement and Vitality (B = 0.326, 95% CI [0.220, 0.432], *p* < 0.001) were statistically significant. Job Satisfaction and Reward also showed the strongest total association with subjective well-being (B = 0.816, 95% CI [0.646, 0.986], *p* < 0.001), supporting H3d as a complementary partial mediation pattern.

Regarding the covariates, age was positively associated with both Work Engagement and Vitality, as well as subjective well-being. Work experience was negatively associated with Work Engagement and Vitality but was not significantly associated with subjective well-being. None of the professional-position categories differed significantly from physicians in either Work Engagement and Vitality or subjective well-being. Therefore, adjustment for professional role did not materially alter the principal structural and indirect associations.

Detailed estimates for all direct, indirect, and total associations, covariate coefficients, and model-fit statistics are provided in Supplementary Material File S3. Given the cross-sectional design, the reported indirect associations should be interpreted as statistical pathways rather than evidence of causal mediation.

### 3.5. Results of the Female-Only Sensitivity Analysis

Given the marked predominance of women in the study sample, a sensitivity analysis was conducted by re-estimating the comprehensive multiple-predictor mediation SEM in the female subsample. Of the 509 female participants included in the descriptive sample, four had missing values for one or more covariates. Because complete-case analysis was used, these cases were excluded, resulting in a final analytical sample of 505 female healthcare professionals. All four psychosocial work environment factors were included simultaneously, with Work Engagement and Vitality specified as the mediator and subjective well-being as the outcome. The model was adjusted for age, work experience, and professional position, with physicians used as the reference category.

The female-only model demonstrated good fit: χ^2^(951) = 2363.16, *p* < 0.001; CFI = 0.946; TLI = 0.955; RMSEA = 0.054, 95% CI [0.052, 0.057]; and SRMR = 0.065. The model explained 65.0% of the variance in Work Engagement and Vitality and 56.7% of the variance in subjective well-being. Work Engagement and Vitality were positively associated with subjective well-being (β = 0.488, *p* < 0.001).

Occupational Distress and Strain showed a significant negative indirect association with subjective well-being through Work Engagement and Vitality (B = −0.180, 95% CI [−0.257, −0.104], *p* < 0.001). Its direct association was not statistically significant, whereas the total association remained negative and significant. Autonomy and Work Control also showed a significant positive indirect association (B = 0.161, 95% CI [0.079, 0.243], *p* < 0.001), while its direct association was not statistically significant.

Job Satisfaction and Reward demonstrated both a significant positive direct association (B = 0.430, 95% CI [0.258, 0.603], *p* < 0.001) and a significant positive indirect association through Work Engagement and Vitality (B = 0.373, 95% CI [0.247, 0.498], *p* < 0.001). It also showed the strongest total association with subjective well-being (B = 0.803, 95% CI [0.626, 0.980], *p* < 0.001).

Organizational Trust and Support showed a significant positive indirect association (B = 0.253, 95% CI [0.098, 0.408], *p* = 0.001) but a significant negative direct association (B = −0.525, 95% CI [−0.826, −0.224], *p* < 0.001). Its total association was not statistically significant, indicating an inconsistent pattern that should be interpreted cautiously.

Among the covariates, age was positively associated with both Work Engagement and Vitality and subjective well-being, whereas work experience was negatively associated with Work Engagement and Vitality but not significantly associated with subjective well-being. General care nurses reported higher Work Engagement and Vitality than physicians, while the remaining professional-position comparisons were not statistically significant.

Overall, the female-only sensitivity analysis supported the principal statistical indirect associations observed for Occupational Distress and Strain, Autonomy and Work Control, and Job Satisfaction and Reward. The results for Organizational Trust and Support remained inconsistent. Detailed path estimates, covariate associations, and model-fit information for the female-only sensitivity analysis are presented in Supplementary Materials File S4. Given the cross-sectional study design, these findings represent statistical indirect associations and should not be interpreted as evidence of causal mediation.

## 4. Discussion

This study examined the associations between psychosocial work environment factors and subjective well-being among healthcare professionals, with particular attention to both direct and indirect pathways. Using structural equation modeling, the study tested whether job demands and job resources were associated with subjective well-being and whether Work Engagement and Vitality functioned as an indirect pathway linking selected environmental factors to well-being.

### 4.1. Direct and Total Associations of Psychosocial Work Environment Factors with Subjective Well-Being

In the comprehensive multiple-predictor model, Job Satisfaction and Reward was the only psychosocial factor showing a statistically significant direct association with subjective well-being in the hypothesized direction. This finding supports H2c and indicates that perceived recognition, reward, and satisfaction with work remained independently associated with subjective well-being after simultaneous adjustment for the other psychosocial factors and covariates [13,18].

The direct associations of Occupational Distress and Strain and Autonomy and Work Control with subjective well-being were not statistically significant in the comprehensive model. Therefore, H1 and H2b were not supported at the direct-path level. However, their total associations remained statistically significant in the expected negative and positive directions, respectively, primarily because of the statistical indirect associations through Work Engagement and Vitality. These findings suggest that their relationships with subjective well-being may be more accurately represented by the combined direct and indirect pathways than by the direct paths alone [13,14,16].

Organizational Trust and Support showed a statistically significant negative direct association with subjective well-being, which was opposite to the hypothesized direction. At the same time, its indirect association through Work Engagement and Vitality was positive, and its total association was not statistically significant. Therefore, H2a was not supported, and the observed pattern should be interpreted as inconsistent mediation rather than as evidence of a straightforward beneficial or harmful association [14,18].

### 4.2. Mediating Role of Work Engagement and Vitality

The comprehensive multiple-predictor mediation model provided additional insight into the statistical indirect associations between psychosocial work environment factors and subjective well-being. Although Work Engagement and Vitality were not significantly associated with subjective well-being at the bivariate level, they were retained as the proposed mediators because their roles had been specified a priori on the basis of the Job Demands–Resources framework and previous empirical evidence [13,14].

A statistically significant zero-order association between a proposed mediator and an outcome is not a necessary prerequisite for testing an indirect association. Indirect associations are estimated from the product of the predictor-to-mediator and mediator-to-outcome paths and may be statistically significant even when the corresponding bivariate association is weak or non-significant, particularly when several correlated predictors are included simultaneously or when direct and indirect pathways operate in different directions [34]. Nevertheless, the absence of a significant bivariate association between Work Engagement and Vitality and subjective well-being limits the strength of conclusions regarding its mediating role and requires cautious interpretation.

Occupational Distress and Strain showed a significant negative association with Work Engagement and Vitality and a significant negative indirect association with subjective well-being. Its direct association with subjective well-being was not statistically significant after simultaneous adjustment for the other psychosocial predictors and covariates, whereas its total association remained negative and significant. This pattern is consistent with a statistical indirect-only association and supports H3a. The direction of the association was consistent with the health impairment process proposed by the JD-R model, according to which excessive occupational demands and strain may be associated with reduced energetic and motivational resources and, subsequently, lower subjective well-being [13,16]. However, the cross-sectional design does not establish that occupational strain temporally preceded lower engagement or well-being.

Autonomy and Work Control also demonstrated a significant positive association with Work Engagement and Vitality and a significant positive indirect association with subjective well-being. Its direct association with subjective well-being was not statistically significant, whereas the total association remained positive and significant. This pattern supports H3c as a statistical indirect-only association. The findings suggest that autonomy and perceived control may be associated with subjective well-being primarily through greater professional involvement, energy, and vitality. This interpretation is consistent with the JD-R model, which identifies autonomy and decision latitude as important job resources supporting motivation and positive work-related functioning [13,14]. Nevertheless, this result should be interpreted cautiously because of the limited convergent validity and internal consistency of the Autonomy and Work Control construct.

Job Satisfaction and Reward demonstrated the strongest positive association with Work Engagement and Vitality. Both its direct association with subjective well-being and its indirect association through Work Engagement and Vitality were statistically significant, and it showed the strongest total association with subjective well-being. This complementary partial mediation pattern supports H3d. The findings indicate that satisfaction, recognition, and perceived reward may be associated with subjective well-being both directly, through positive evaluations of work, and indirectly, through higher levels of engagement and vitality. This pattern is consistent with the motivational process proposed by the JD-R model, in which job resources are associated with greater motivation and more positive psychological outcomes [13,14,18].

The pattern observed for Organizational Trust and Support was more complex. Organizational Trust and Support was positively associated with Work Engagement and Vitality and showed a significant positive indirect association with subjective well-being. However, its direct association with subjective well-being was negative, while its total association was not statistically significant. The opposing direct and indirect pathways indicate inconsistent mediation and do not provide clear support for H3b. This finding should not be interpreted as evidence that organizational trust and support has a straightforward beneficial or harmful association with subjective well-being. Instead, it may reflect statistical suppression, overlap with other job resources included in the comprehensive model, or unmeasured organizational processes. Therefore, this result should be considered exploratory and requires replication in an independently recruited sample.

Taken together, the findings indicate that Work Engagement and Vitality may represent a statistical indirect pathway linking selected psychosocial work environment factors with subjective well-being. The theoretically coherent negative indirect association for Occupational Distress and Strain and the positive indirect associations for Autonomy and Work Control and Job Satisfaction and Reward were supported. In contrast, the pattern involving Organizational Trust and Support remained inconsistent and should be interpreted cautiously. Because the data were cross-sectional, the observed indirect associations do not establish temporal ordering or causal mediation. Longitudinal studies with repeated measurements are needed to determine whether changes in psychosocial work environment factors precede changes in Work Engagement and Vitality and subsequent changes in subjective well-being.

### 4.3. Interpretation of the Female-Only Sensitivity Analysis

Given the marked predominance of women in the study sample, an additional sensitivity analysis was conducted among female healthcare professionals to assess whether the principal findings remained stable within the largest gender subgroup. The female-only comprehensive mediation model demonstrated good fit and explained a substantial proportion of the variance in both Work Engagement and Vitality and subjective well-being. These findings indicate that the overall pattern of associations was not solely attributable to the inclusion of the relatively small number of male participants.

Within the female subsample, Job Satisfaction and Reward remained the most prominent psychosocial resource associated with subjective well-being. It demonstrated both significant direct and indirect associations through Work Engagement and Vitality, suggesting that recognition, perceived reward, and positive evaluations of work may be particularly relevant to the well-being of female healthcare professionals. This pattern was consistent with the findings obtained in the full sample and supports the motivational pathway proposed by the JD-R framework, according to which job resources may foster engagement and positive psychological outcomes [13,14,18].

Autonomy and Work Control demonstrated a significant positive indirect association with subjective well-being through Work Engagement and Vitality, whereas its direct association was not statistically significant. This finding suggests that, among women, autonomy may be associated with well-being primarily through greater professional involvement, energy, and vitality. This interpretation is consistent with the JD-R model, which conceptualizes autonomy and decision latitude as important job resources supporting motivation and positive work-related functioning [13,14]. However, given the limited measurement quality of the Autonomy and Work Control construct, this finding should be interpreted cautiously and requires confirmation in an independent sample.

Occupational Distress and Strain showed a significant negative indirect association with subjective well-being through Work Engagement and Vitality. In contrast to the paradoxical pattern observed in the separate mediation model, the direction of this association was theoretically consistent when all psychosocial predictors were included simultaneously. This result is consistent with the health impairment process proposed by the JD-R model, in which excessive demands and occupational strain may deplete employees’ psychological and energetic resources [13,16]. The finding also suggests that controlling for shared variance among job demands and job resources may provide a more accurate representation of the association between occupational strain, engagement, and well-being.

Organizational Trust and Support continued to show an inconsistent pattern, characterized by a positive indirect association through Work Engagement and Vitality but a negative direct association with subjective well-being. Because the total association was not statistically significant, this finding should not be interpreted as evidence of a straightforward beneficial or harmful relationship. Instead, it may reflect statistical suppression, overlap with other job resources, or unmeasured organizational processes. Previous research nevertheless indicates that organizational support and psychosocial resources may contribute to employee engagement and well-being through complex and context-dependent pathways [14,18].

Overall, the female-only sensitivity analysis strengthened confidence in the robustness of the principal associations involving Job Satisfaction and Reward, Autonomy and Work Control, and Occupational Distress and Strain. Nevertheless, this analysis does not demonstrate that the associations differ by gender, because an adequately powered male-only model or formal gender moderation analysis could not be performed. The findings may therefore be considered particularly relevant to female healthcare professionals, while their applicability to male healthcare professionals remains uncertain. As with the primary analysis, the cross-sectional design permits conclusions about statistical associations but not causal or temporal mechanisms [22,23].

### 4.4. Practical Implications

The findings have several practical implications for healthcare organizations and policymakers. First, the strong association between Job Satisfaction and Reward and subjective well-being suggests that healthcare organizations should prioritize fair recognition, meaningful feedback, professional appreciation, and reward systems. These factors may be particularly important in healthcare settings where high responsibility and emotional demands are common.

Second, the positive association between Autonomy and Work Control and subjective well-being highlights the importance of involving healthcare professionals in decision-making processes and providing greater flexibility where possible. Strategies that support professional autonomy, participatory management, and control over work processes may contribute to higher motivation and better well-being.

Third, the negative association between Occupational Distress and Strain and subjective well-being emphasizes the need to reduce excessive job demands. This may include workload management, adequate staffing, emotional support systems, and organizational strategies to prevent chronic strain and exhaustion.

Finally, the mediation findings suggest that interventions should not focus only on structural work environment factors but should also promote engagement, energy, and professional vitality. However, such interventions should be implemented carefully and evaluated empirically, as the present findings indicate that the relationships among work environment factors, engagement, and well-being may be complex.

### 4.5. Limitations

Several limitations should be acknowledged. First, a cross-sectional design limits the ability to draw causal conclusions. Although mediation models were tested, the results represent statistical indirect associations rather than evidence of causal mechanisms. Future longitudinal studies are needed to examine the temporal ordering of work environment factors, Work Engagement and Vitality, and subjective well-being.

Second, the study relied on self-reported data, which may be affected by common-method bias, response tendencies, and social desirability bias. Although self-report measures are widely used in occupational health research, future studies could strengthen the evidence by including objective organizational indicators or multi-source data.

Third, the sample was obtained using a non-probability convenience sampling approach, which may limit the generalizability of the findings and introduce a risk of selection bias. Because the survey invitation was distributed by human resources representatives through institutional work email addresses, internal WhatsApp groups, and other internal communication channels, the exact number of healthcare professionals who received or viewed the invitation was not recorded. Consequently, a conventional response rate could not be calculated, and potential non-response bias could not be directly assessed. Healthcare professionals who chose to participate may have differed systematically from non-respondents in terms of subjective well-being, workload, or perceptions of the psychosocial work environment. Although the sample included several major professional groups, including physicians, nurses, physician assistants, and other healthcare professionals, participants were recruited only from the 13 participating hospitals and were not selected proportionally according to the national distribution of healthcare professions. Therefore, the final sample cannot be considered nationally representative of all healthcare professionals in Latvia, particularly those working in primary care, outpatient settings, long-term care, or institutions not included in the recruitment process. Accordingly, the findings should be generalized to the broader Latvian healthcare workforce with caution. The demographic and professional composition of the sample was not formally compared with national healthcare workforce statistics. Moreover, because the number and professional characteristics of all healthcare professionals who received the invitation were not recorded, it was not possible to determine whether the predominance of women and physicians reflected the workforce composition of the participating hospitals or differences in response patterns. Therefore, the proportion of physicians in the sample should not be interpreted as evidence that physicians constitute the majority of healthcare professionals involved in direct patient care in Latvia.

In addition, the sample was predominantly female, reflecting the gender structure of many healthcare professions but limiting the ability to examine gender differences in detail. No gender-specific recruitment quotas or interim monitoring of the sample’s demographic composition were implemented during data collection; therefore, recruitment strategies were not adjusted to increase male participation. Future studies should use stratified or targeted recruitment strategies and periodically monitor sample demographics to improve gender balance, reduce potential selection bias, and enable more reliable gender-specific analyses.

Fourth, the response option “prefer not to answer/not applicable” was treated as missing and was not included in the scale score calculation or statistical modeling. Although this approach is methodologically appropriate because this response option does not represent a higher level of agreement, missing responses may still influence the precision of estimates.

Fifth, the measurement quality of some HP-PSWB constructs was below conventional psychometric thresholds. In particular, Autonomy and Work Control demonstrated borderline internal consistency (Cronbach’s α = 0.662; CR = 0.644) and limited convergent validity (AVE = 0.328). Although this construct was retained because of its theoretical importance within the JD-R framework and satisfactory discriminant validity, its relatively low reliability and AVE may reduce the precision and stability of the estimated structural associations. Therefore, findings involving Autonomy and Work Control should be interpreted cautiously. Future studies should refine the lower-performing items and independently re-evaluate the construct’s reliability and validity before drawing firm conclusions regarding its association with subjective well-being.

Sixth, although the EFA and CFA were conducted in separate, randomly assigned, non-overlapping subsamples, the subsequent structural equation modeling and mediation analyses were performed using the same overall dataset. Therefore, the measurement and structural models were not externally validated in an independently recruited sample. This may increase the extent to which the estimated associations are specific to the present dataset. Future studies should replicate both the factor structure and the structural associations in an independent sample of healthcare professionals.

Seventh, the mediation findings require cautious interpretation. Work Engagement and Vitality were not significantly correlated with subjective well-being at the bivariate level (r = 0.010, *p* = 0.816). Although a statistically significant zero-order correlation is not a necessary prerequisite for testing an indirect association, the absence of such an association reduces the strength of the empirical support for Work Engagement and Vitality as a mediator in the present sample. The observed indirect associations may be model-dependent and may reflect suppression effects, shared variance among the psychosocial constructs, or alternative model specifications. In particular, the indirect association involving Occupational Distress and Strain showed a direction that differed from the theoretically expected negative pattern. Similarly, the inconsistent mediation pattern observed for Organizational Trust and Support should be regarded as exploratory. Therefore, the mediation findings should not be interpreted as evidence of an established psychological mechanism and require replication using longitudinal data, repeated measurements, and alternative model specifications.

Finally, although the study included several important psychosocial work environment factors, other variables that may influence subjective well-being were not included. Future research could extend the model by incorporating additional individual, organizational, and system-level factors.

## 5. Conclusions

This study demonstrates that psychosocial work environment factors are meaningfully associated with subjective well-being among healthcare professionals. Job Satisfaction and Reward were the strongest positive predictors of subjective well-being, while Autonomy and Work Control also showed a positive association. In contrast, Occupational Distress and Strain were negatively associated with subjective well-being, highlighting the detrimental role of work-related demands and strain.

The findings also suggest that Work Engagement and Vitality may function as an important indirect pathway linking selected psychosocial work environment factors to subjective well-being. The most theoretically consistent indirect associations were observed between Autonomy and Work Control, and between Job Satisfaction and Reward. However, some mediation patterns were complex and should be interpreted cautiously, particularly given the cross-sectional design.

Overall, the study contributes to a better understanding of how job demands and job resources are associated with subjective well-being in healthcare settings. The findings underscore the relevance of the JD-R model and the importance of improving conditions for reward, autonomy, and workload to support the well-being of healthcare professionals.

## Figures and Tables

**Table 1:** EFA Pattern Matrix, Communalities, and Reliability Indices for the Refined 35-Item HP-PSWB Scale.

Factor and Items	Loading	h^2^	α	ω
Factor 1: Job Satisfaction and Reward			0.911	0.915
A30. Satisfied with cooperation	0.82	0.65		
A26. Satisfied with current job	0.78	0.63		
A28. Contribution is appreciated	0.76	0.64		
A31. Satisfied with salary	0.75	0.69		
A27. Work is rewarding	0.67	0.38		
A29. Conditions allow effective work	0.67	0.63		
A21. Negative physical impact	0.67	0.57		
A25. Feeling full of energy	0.56	0.47		
A15. No work-life conflict	0.53	0.44		
Factor 2: Work Engagement and Vitality			0.887	0.889
A34. Engaged in professional tasks	0.81	0.56		
A18. Managing workload efficiently	0.79	0.66		
A33. Inspired and enthusiastic	0.76	0.72		
A5 Maintain optimism	0.61	0.42		
A17. Pace of work is acceptable	0.58	0.54		
A35. Apply knowledge and skills	0.57	0.35		
A6 Recover quickly after stress	0.54	0.41		
A32. Full of energy	0.52	0.6		
Factor 3: Occupational Distress and Strain			0.843	0.852
A20. Emotional exhaustion	0.79	0.64		
A19. Physical strain	0.64	0.58		
A16. Emotional demands	0.54	0.36		
A22. Health issues	0.64	0.38		
A23. Burnout symptoms	0.64	0.49		
A24. Cynicism	0.53	0.42		
Factor 4: Organizational Trust and Support			0.829	0.832
A3. Supervisor support	0.85	0.73		
A4. Management respect	0.84	0.7		
A2. Trust in management	0.74	0.56		
A12. Professional opinion valued	0.46	0.45		
A14. Training availability	0.42	0.28		
A13. Support for education	0.40	0.32		
A1. Trust in colleagues	0.40	0.19		
Factor 5: Autonomy and Work Control			0.758	0.765
A8. Influence workload	0.68	0.58		
A9. Plan working day	0.56	0.46		
A7. Decision-making participation	0.43	0.39		
A11. Choose work methods	0.43	0.32		
A10. Autonomy in tasks	0.42	0.31		

Note. EFA = exploratory factor analysis; *h*^2^ = communality; α = Cronbach’s alpha; ω = McDonald’s omega. The EFA was conducted in the randomly selected subsample (*n* = 260). Only the primary factor loading for each retained item is presented. Cronbach’s α and McDonald’s ω are reported at the factor level. Reverse-worded items were recoded before the analysis.

**Table 2:** Standardized CFA Factor Loadings for the Refined 35-Item HP-PSWB Scale.

Latent Construct and Items	Standardized Loading	α	CR	AVE
Job Satisfaction and Reward		0.91	0.917	0.591
A30. Satisfied with cooperation	0.830			
A31. Satisfied with salary	0.823			
A29. Conditions allow effective work	0.808			
A25. Feeling full of energy	0.776			
A26. Satisfied with current job	0.774			
A28. Contribution is appreciated	0.772			
A21. Negative physical impact	0.742			
A27. Work is rewarding	0.71			
A15. No work-life conflict	0.669			
Work Engagement and Vitality		0.832	0.863	0.458
A33. Inspired and enthusiastic	0.819			
A32. Full of energy	0.808			
A17. Pace of work is acceptable	0.741			
A18. Managing workload efficiently	0.618			
A6. Recover quickly after stress	0.615			
A34. Engaged in professional tasks	0.61			
A5 Maintain optimism	0.582			
A35. Apply knowledge and skills	0.568			
Occupational Distress and Strain		0.79	0.763	0.465
A19. Physical strain	0.847			
A20. Emotional exhaustion	0.842			
A24. Cynicism	0.765			
A23. Burnout symptoms	0.728			
A22. Health issues	0.409			
A16. Emotional demands	0.409			
Organizational Trust and Support		0.775	0.738	0.363
A2. Trust in management	0.735			
A13. Support for education	0.712			
A3. Supervisor support	0.686			
A14. Training availability	0.673			
A4. Management respect	0.669			
A12. Professional opinion valued	0.659			
A1. Trust in colleagues	0.463			
Autonomy and Work Control		0.662	0.644	0.328
A9. Plan for a working day	0.839			
A8. Influence workload	0.616			
A11. Choose work methods	0.551			
A7 Decision-making participation	0.494			
A10. Autonomy in tasks	0.481			

Note. CFA = confirmatory factor analysis; α = Cronbach’s alpha; CR = composite reliability; AVE = average variance extracted. The CFA was conducted in a separate, randomly selected, non-overlapping subsample (*n* = 306) using the WLSMV estimator. Standardized factor loadings are reported at the item level, whereas α, CR, and AVE are reported at the latent-construct level. Reverse-worded items were recoded before the analysis.

**Table 3:** Sample characteristics (*N* = 566).

Variable	*n* (%)/Mean (SD)
Age	48.59 (12.87)
Work experience (years)	22.53 (14.30)
Gender	
Female	509 (89.9%)
Male	48 (8.5%)
Prefer not to disclose	9 (1.6%)
Healthcare institution type	
Public healthcare institution	280 (49.5%)
Municipal healthcare institution	157 (27.7%)
Private healthcare institution	72 (12.7%)
Other	57 (10.1%)
Professional position	
Physician	242 (42.8%)
Resident physician	37 (6.5%)
Physician assistant	47 (8.3%)
Nurse	173 (30.6%)
Other	67 (11.8%)
Workload	
≤0.25 full-time equivalent	5 (0.9%)
0.5 full-time equivalent	28 (4.9%)
0.75 full-time equivalent	28 (4.9%)
1.0 full-time equivalent	373 (65.9%)
>1.0 full-time equivalent	107 (18.9%)
Other	25 (4.5%)
Education	
Short-cycle higher education degree	179 (31.6%)
Medical degree	232 (41.0%)
Postgraduate medical residency training	40 (7.1%)
Doctoral degree	8 (1.4%)
Other or unspecified education	107 (18.9%)

Note. Values are presented as mean (SD) or frequency (*n*, %).

**Table 4:** Descriptive Statistics and Normality Tests (*N* = 566).

Variable	*N*	Median	IQR	Skewness	Kurtosis	Shapiro–Wilk (*p*)
Subjective Well-Being	566	16.00	7.00	0.020	−0.664	<0.001
Job Satisfaction and Reward	566	3.13	1.13	0.256	−0.754	<0.001
Work Engagement and Vitality	566	3.13	0.88	−0.346	−0.105	<0.001
Occupational Distress and Strain	566	2.83	1.00	0.463	−0.389	<0.001
Organizational Trust and Support	566	2.64	1.00	0.453	−0.391	<0.001
Autonomy and Work Control	566	3.00	1.00	−0.167	−0.132	<0.001

Note. All Shapiro–Wilk tests were statistically significant (*p* < 0.001), indicating deviation from a normal distribution.

**Table 5:** Spearman Correlations Between Study Variables.

Variable	1	2	3	4	5	6
Job Satisfaction and Reward	—					
Work Engagement and Vitality	0.041	—				
Occupational Distress and Strain	−0.500 ***	0.054	—			
Organizational Trust and Support	0.516 ***	0.052	−0.451 ***	—		
Autonomy and Work Control	0.589 ***	−0.023	−0.521 ***	0.416 ***	—	
Subjective Well-Being	0.521 ***	0.010	−0.442 ***	0.451 ***	0.512 ***	—

Note. Spearman’s rho correlation coefficients are reported. Variables: (1) Job Satisfaction and Reward, (2) Work Engagement and Vitality, (3) Occupational Distress and Strain, (4) Organizational Trust and Support, (5) Autonomy and Work Control, and (6) Subjective Well-Being. *** *p* < 0.001.

## Data Availability

The data supporting the findings of this study are available from the corresponding author upon reasonable request. The data are not publicly available due to ethical and data protection considerations.

## Supplementary Materials

The following supporting information can be downloaded at: https://www.mdpi.com/article/10.3390/ijerph23080976/s1. File S1: English Version of the Healthcare Professionals Psychosocial Well-Being Scale (HP-PSWB); File S2: Latvian Version of the Healthcare Professionals Psychosocial Well-Being Scale (HP-PSWB); File S3: Detailed Results of the Comprehensive Multiple-Predictor Mediation SEM in the Full Sample; File S4: Detailed Results of the Female-Only Sensitivity Analysis.

## Abbreviations

AVE	Average Variance Extracted
CFA	Confirmatory Factor Analysis
CFI	Comparative Fit Index
CI	Confidence Interval
CR	Composite Reliability
CSRD	Corporate Sustainability Reporting Directive
EFA	Exploratory Factor Analysis
GDPR	General Data Protection Regulation
HP-PSWB	Healthcare Professionals Psychosocial Well-Being Scale
HTMT	Heterotrait–Monotrait Ratio of Correlations
IQR	Interquartile Range
JD-R	Job Demands–Resources
OECD	Organization for Economic Co-operation and Development
RMSEA	Root Mean Square Error of Approximation
SEM	Structural Equation Modeling
SRMR	Standardized Root Mean Square Residual
SWB	Subjective Well-Being
TLI	Tucker–Lewis Index
WHO	World Health Organization
WHO-5	World Health Organization Five Well-Being Index
WLSMV	Weighted Least Squares Mean and Variance Adjusted Estimator
