# Supplementary material for: Work Environment Factors and Subjective Well-Being Among Healthcare Professionals: A Structural Equation Modeling Study with Mediation Analysis
Source: Int J Environ Res Public Health. 2026 Jul 28;23(8):976. doi: 10.3390/ijerph23080976 (PMC13512546; doi:10.3390/ijerph23080976)
Supplement: Supplementary file 1 [file ijerph-23-00976-s001.zip › ijerph-4426042-supplementary/File S1.pdf]

Healthcare Professionals Psychosocial Well-Being Scale (HP-PSWB)

This questionnaire asks about your healthcare experiences. It focuses on your work environment, job demands, available resources, and overall well-being.

In this questionnaire, "patient" means those who receive care from you. This can also include a client or service user, depending on your job.

Indicate how true each statement is for you based on your work experience. There are no right or wrong answers. Your honest opinion and personal experience matter.

For each statement, select the response that best matches your experience. When possible, give your first impression without overthinking.

1	2	3	4	5
Strongly disagree	Somewhat disagree	Somewhat agree	Agree	Prefer not to answer / Not applicable

1	I trust my colleagues.	1	2	3	4	5
2	I trust the hospital management.	1	2	3	4	5
3	My supervisor supports me in professional matters.	1	2	3	4	5
4	My management treats me with respect.	1	2	3	4	5
5	Even in difficult situations, I can maintain optimism.	1	2	3	4	5
6	I can recover quickly after stressful situations at work.	1	2	3	4	5
7	I can participate in decision-making regarding the organization of my department's work.	1	2	3	4	5
8	I can influence my workload.	1	2	3	4	5
9	I can plan my working day.	1	2	3	4	5
10	I can influence my work schedule.	1	2	3	4	5
11	I can openly point out mistakes in the care process.	1	2	3	4	5
12	I feel safe expressing suggestions or objections to senior colleagues.	1	2	3	4	5
13	During the past year, I have received training that improved my future career opportunities.	1	2	3	4	5
14	During the past year, I have received training that improved my professional skills.	1	2	3	4	5
15R	After work, I often do not have enough time to rest and recover at home.	1	2	3	4	5
16R	I often must communicate with patients' families about serious situations.	1	2	3	4	5
17R	The amount of work I am responsible for often exceeds what can be done during normal working hours.	1	2	3	4	5
18R	Due to workload, it is often difficult for me to complete tasks with the expected quality, and my shift lasts longer than planned.	1	2	3	4	5
19R	At the end of the workday, I feel physically exhausted and find it difficult to recover.	1	2	3	4	5
20R	My work involves lifting or moving heavy objects (including people).	1	2	3	4	5
21R	My work has negatively affected my physical health.	1	2	3	4	5
22R	During the past 12 months, I have experienced symptoms related to my physical health.	1	2	3	4	5
23R	My work emotionally exhausts me.	1	2	3	4	5
24R	My work makes me feel tired already in the morning.	1	2	3	4	5
25R	After work, I feel too tired to do other activities.	1	2	3	4	5
26	I am satisfied with my current job.	1	2	3	4	5
27	I find my work rewarding.	1	2	3	4	5
28	I feel that my work contribution is appreciated.	1	2	3	4	5
29	My working conditions allow me to perform my job effectively.	1	2	3	4	5
30	I am satisfied with the cooperation with my colleagues.	1	2	3	4	5
31	I am satisfied with the salary in my job.	1	2	3	4	5
32	At work, I feel full of energy.	1	2	3	4	5
33	At work, I feel inspired and enthusiastic.	1	2	3	4	5
34	I am proud of the work I do.	1	2	3	4	5
35	In my job, I can apply my knowledge and skills.	1	2	3	4	5

Thank you for completing this questionnaire.

**Psychosocial construct**	**Item numbers**	**Number of items (n)**	**Reverse-coded items**
**Factor 1: Job Satisfaction and Reward**	15, 21, 25, 26, 27, 28, 29, 30, 31	9	15, 21, 25
**Factor 2: Work Engagement and Vitality**	5, 6, 17, 18, 32, 33, 34, 35	8	17, 18
**Factor 3: Occupational Distress and Strain**	16, 19, 20, 22, 23, 24	6	16, 19, 20, 22, 23, 24
**Factor 4: Organizational Trust and Support**	1, 2, 3, 4, 12, 13, 14	7	-
**Factor 5: Autonomy and Control**	7, 8, 9, 10, 11	5	-
