# Supplementary material for: Work Environment Factors and Subjective Well-Being Among Healthcare Professionals: A Structural Equation Modeling Study with Mediation Analysis
Source: Int J Environ Res Public Health. 2026 Jul 28;23(8):976. doi: 10.3390/ijerph23080976 (PMC13512546; doi:10.3390/ijerph23080976)
Supplement: Supplementary file 1 [file ijerph-23-00976-s001.zip › ijerph-4426042-supplementary/File S2.pdf]

Ārstniecības personu psihosociālās subjektīvās labbūtības skala

Cienījamais dalībniek!

Zemāk ir doti vairāki apgalvojumi par to, kā cilvēki mēdz justies ikdienā darba vietā. Lūdzu, atzīmējiet vienu atbildi katrā rindā. Lūdzu atbildiet par savu pamatdarbu, kur Jums ir lielāka slodze vai lielāka alga, ja ir vairākas darbavietas.

Lūdzu, rūpīgi izlasiet katru apgalvojumu/jautājumu un novērtējiet, cik lielā mērā piekrītat katram apgalvojumam. Pirms katra jautājumu bloka lietojiet norādīto vērtējumu skalu.

1	2	3	4	5
**Nepiekrītu**	**Drīzāk nepiekrītu**	**Drīzāk piekrītu**	**Piekrītu**	**Nevēlos atbildēt/Neattiecas**

Aptauja neparedz pareizās vai nepareizās atbildes

1	Es uzticos saviem kolēģiem	1	2	3	4	5
2	Es uzticos slimnīcas vadībai	1	2	3	4	5
3	Mans vadītājs mani atbalsta profesionālos jautājumos	1	2	3	4	5
4	Mana vadība izturas ar cieņu pret mani	1	2	3	4	5
5	Pat grūtās situācijās es spēju saglabāt optimismu	1	2	3	4	5
6	Es spēju ātri atgūties pēc stresa situācijām darbā	1	2	3	4	5
7	Es varu iesaistīties lēmumu pieņemšanā par nodaļas darba organizāciju	1	2	3	4	5
8	Es varu ietekmēt savu darba apjomu	1	2	3	4	5
9	Es varu plānot savu darba dienu	1	2	3	4	5
10	Es varu ietekmēt savu darba grafiku	1	2	3	4	5
11	Es varu atklāti norādīt uz kļūdām aprūpes procesā	1	2	3	4	5
12	Es jūtos droši izteikt ieteikumus/ iebildumus augstākstāvošajiem kolēģiem	1	2	3	4	5
13	Pēdējā gada laikā esmu saņēmis/-usi mācības kas uzlabo manas nākotnes darba iespējas	1	2	3	4	5
14	Pēdējā gada laikā esmu saņēmis/-usi mācības kas ir uzlabojušas manas prasmes	1	2	3	4	5
15	Pēc darba man trūkst laika atpūtai un atjaunošanās iespējām mājās	1	2	3	4	5
16	Bieži jākomunicē ar pacientu ģimenēm par smagām situācijām	1	2	3	4	5
17	Darba pienākumu apjoms bieži pārsniedz to, ko var paveikt normālā darba laikā	1	2	3	4	5
18	Pārslodzes dēļ man ir grūti kvalitatīvi pildīt uzdevumus, un darba maiņa ieilgst ilgāk nekā plānots	1	2	3	4	5
19	Darba dienas beigās es izjūtu fizisku nogurumu un grūtības atjaunoties	1	2	3	4	5
20	Mans darbs ir saistīts ar smagumu (tai skaitā cilvēku) celšanu, pārvietošanu	1	2	3	4	5
21	Darbs ir negatīvi ietekmējis manu fizisko veselību	1	2	3	4	5
22	Pēdējo 12 mēnešu laikā esat sev novērojis/usi fiziskās veselības simptomus	1	2	3	4	5
23	Mans darbs emocionāli mani izsmeļ	1	2	3	4	5
24	Mans darbs liek man justies nogurušam(-ai) jau no paša rīta	1	2	3	4	5
25	Pēc darba es jūtos pārāk noguris(-usi), lai darītu citas lietas	1	2	3	4	5
26	Es jūtos apmierināts(-a) ar savu pašreizējo darbu	1	2	3	4	5
27	Es gūstu gandarījumu savā darbā	1	2	3	4	5
28	Es jūtu, ka mana darba ieguldījums tiek novērtēts	1	2	3	4	5
29	Darba apstākļi man ļauj kvalitatīvi veikt darbu	1	2	3	4	5
30	Es esmu apmierināts(-a) ar sadarbību ar saviem kolēģiem	1	2	3	4	5
31	Es esmu apmierināts(-a) ar atalgojumu savā darbā	1	2	3	4	5
32	Darba laikā jūtos enerģijas pilns(-a)	1	2	3	4	5
33	Darba vietā jūtos iedvesmots(-a) un entuziasma pilns(-a)	1	2	3	4	5
34	Esmu lepns(-a) par darbu, ko daru	1	2	3	4	5
35	Savā darbā es varu pielietot savas zināšanas un prasmes	1	2	3	4	5

Paldies par dalību!

Psihosociālais konstrukts	Vienumu numuri	Vienumu skaits (n)	Apgrieztie vienumi
**1. faktors: Darba apmierinātība un atalgojums**	15, 21, 25, 26, 27, 28, 29, 30, 31	9	15, 21, 25
**2. faktors: Darba iesaiste un vitalitāte**	5, 6, 17, 18, 32, 33, 34, 35	8	17,18
**3. faktors: Darba spriedze un distress**	16, 19, 20, 22, 23, 24	6	16, 19, 20, 22, 23, 24
**4. faktors: Organizācijas uzticēšanās un atbalsts**	1, 2, 3, 4, 12, 13, 14	7	-
**5. faktors: Autonomija un kontrole**	7, 8, 9, 10, 11	5	-
